# Clinical characteristics and therapeutic effects of checkpoint inhibitor-related pneumonitis in patients with non-small cell lung cancer

**DOI:** 10.1186/s12885-023-10649-0

**Published:** 2023-03-03

**Authors:** Li Pang, Mei Xie, Xidong Ma, Aiben Huang, Jialin Song, Jie Yao, Hui Deng, Duchao Zhang, Xuelei Zang, Fangping Ren, Jie Gao, Chongchong Wu, Yuanyong Wang, Xin Zhang, Xinyu Bao, Lei Pan, Xinying Xue

**Affiliations:** 1 Department of Respiratory and Critical Care, Beijing Shijitan Hospital, Capital Medical University, 100038 Beijing, China; 2 Department of Respiratory and Critical Care, Chinese PLA General Hospital, 100835 Beijing, China; 3 Department of Respiratory and Critical Care, Weifang Medical College, 261053 Weifang, Shandong China; 4 Department of Pathology, Chinese PLA General Hospital, 100835 Beijing, China; 5 Department of Radiology, Chinese PLA General Hospital, 100835 Beijing, China; 6 Department of Thoracic Surgery, Tangdu Hospital of Air Force Military Medical University, 710038 Xi’an, Shanxi China

**Keywords:** NSCLC, Immune checkpoint inhibitors, Checkpoint inhibitor-associated pneumonia, Immunotherapy

## Abstract

**Background:**

With the application of immune checkpoint inhibitors (ICIs) in cancer treatment, more and more attention has been paid to checkpoint inhibitor-related pneumonitis (CIP), which requires a better understanding of its clinical characteristics and therapeutic effects.

**Methods:**

The clinical and imaging data of 704 patients with non-small cell lung cancer (NSCLC) who received immunotherapy were analyzed retrospectively; the clinical characteristics of CIP were summarized, and the therapeutic regimens and effects of the patients were summarized.

**Results:**

36 CIP patients were included in the research. The most common clinical symptoms were cough, shortness of breath and fever. The CT manifestations were summarized as follows: Organizing pneumonia (OP) in 14 cases (38.9%), nonspecific interstitial pneumonia (NSIP) in 14 cases (38.9%), hypersensitiviy pneumonitis(HP) in 2 cases (6.3%), diffuse alveolar damage in 1 case (3.1%) and atypical imaging manifestations in 5 cases (13.9%). 35 cases received glucocorticoid therapy, 6 patients were treated with gamma globulin and 1 patient was treated with tocilizumab. There were no deaths in CIP G1-2 patients and 7 deaths occured in CIP G3-4 patients. 4 patients were treated again with ICIs.

**Conclusion:**

We found that glucocorticoid 1–2 mg/kg was effective for most patients with moderate to severe CIP, and a few patients with hormone insensitivity needed early immunosuppressive therapy. A few patients can be rechallenged with ICIs, but CIP recurrence needs to be closely monitored.

## Introduction

With the successful application of Immune checkpoint inhibitors (ICIs) in the treatment of melanoma, more and more cancer treatment regimens are incorporating ICIs [1]. At present, in the treatment of non-operative locally advanced and metastatic lung cancer, the application of ICIs alone or in combination with chemotherapy has become the first-line treatment [2]. However, with the continuous expansion of indications of ICIs and its first-line clinical treatment, the immuno-related adverse reactions of the drugs have become more and more common, resulting in multi-organ involvement including the skin, pituitary, thyroid, liver, kidney, lung and other organs, and even life-threatening situations [3]. checkpoint inhibitor-related pneumonitis (CIP) is defined as new or progressive symptoms of dyspnea, cough, chest pain, fever, and fatigue along with new pulmonary exudates in patients who have been treated with ICIs, except for imaging abnormalities caused by pulmonary infection, cancer progression, and other lung diseases [4]. CIP is one of the most common lethal immune-related adverse events (irAEs) that can occur at any time during cancer immunotherapy [5]. Single-agent programmed cell death receptor-1 (PD-1) and PD-L1 inhibitors were the most commonly used ICIs, followed by cytotoxic T lymphocyte-associated protein-4 (CTLA-4). The incidence of CIP in non-small cell lung cancer (NSCLC) is higher than that of most other cancers, and CIP is a common cause of death in cancer patients [6–11]. Different from other interstitial pneumonia, CIP has its special clinical manifestations and imaging characteristics due to its different pathogenesis [5, 12]. However, at present, CIP-related diagnosis and treatment regimens are mostly based on regimens or clinical trial data of interstitial pneumonia. The retrospective study on the diagnosis of CIP and the use of other drugs such as hormones in the real world can better guide clinical treatment.

In this paper, we review and analyze the clinical manifestations, pulmonary imagings and treatment regimens of 36 cases of locally advanced and metastatic NSCLC patients complicated with CIP, summarize the characteristics of these patients and the application of glucocorticoids, which is helpful to guide clinicians in the early diagnosis and treatment of CIP.

## Materials and methods

### Research object

We conducted a retrospective analysis of the clinical data of 704 NSCLC patients treated with ICIs at Beijing Shijitan Hospital, Capital Medical University and Chinese PLA General Hospital (301 Hospital) from January 2016 to December 2019.

Inclusion criteria: Patients with locally advanced or metastatic NSCLC who were confirmed by pathological and imaging data and were treated with ICIs (including PD-1 / PD-L1 inhibitors, CTLA4) ; new imaging abnormalities emerge after treatment with ICIs; CIP was diagnosed by multidisciplinary discussion.

Exclusion criteria: Patients participating in double-blind clinical trials; patients with imaging abnormabilties which were not certainly caused by ICI drugs; imaging abnormalities certainly caused by lung infections; imaging abnormalities caused by cancer progression; previous cases complicated with interstitial pneumonia; patients who were lost to follow-up or with incomplete medical records.

The research was approved by the Hospital Ethics Committee.

## Methods

The diagnosis of 704 cases of NSCLC was made on the basis of postoperative histopathology, CT-guided lung biopsy or bronchoscopic bronchial mucosal biopsy. The clinical data of the patients included their sex, age, smoking history, underlying health conditions, allergy history, tumor location, pathological type, differentiation degree, clinical stage, clinical symptoms, treatment methods and prognosis. The CT data of 704 cases were obtained from the Picture Archiving and Communication Systems (PACS). CT findings of Interstitial lung disease (ILD)were ranged according to the American Thoracic Society/European Respiratory Society (ATS/ERS) international multidisciplinary classification of IP as diffuse alveolar damage (DAD)-like pattern, hypersensitiviy pneumonitis (HP)-like pattern, Organizing pneumonia (OP)-like pattern, nonspecific interstitial pneumonia (NSIP)-like pattern, and others. The chest CT images were inspected by a thoracic radiologist (Chongchong Wu) and a pulmonologist (Hui Deng).

CIP grading creteria: National Comprehensive Cancer Network (NCCN) guidelines grade CIP based on clinical data and imaging manifestations [13], specifically : Grade 1: Asymptomatic, confined to one lobe of the lung or < 25% of lung parenchyma; Grade 2: New respiratory symptoms or exacerbation of the original symptoms, including shortness of breath, cough, chest pain, fever, and increasing oxygen requirements; Grade 3: Severe symptoms, involving all lung lobes or > 50% of lung parenchyma, limiting activities of daily living; Grade 4: Life-threatening difficulty in breathing.

The diagnosis of CIP was determined by the treating oncologist (Mei Xie) and confirmed by a multidisciplinary irAE team consisting of a pulmonologist (Li Pang), radiologist (Chongchong Wu) and a second oncologist (Jialin Song).

### Statistical methods

The time of onset of CIP is defined as the time from the first dose of ICI to the first occurrence of CIP-related symptoms or imaging manifestations in asymptomatic patients.The data is expressed as n (%) for the classified variable and as mean ± standard deviation for the continuous variable. Measurement data was analyzed by chi-square method. Karegimen-Meier was used to analyze the survival of patients with CIP G1-2 and G3-4. A p-value less than 0.05 is statistically significant. SPSS software (Version 25.0; IBM) was used for statistical analysis.

## Results

### Clinical characteristics

**Fig. 1:**
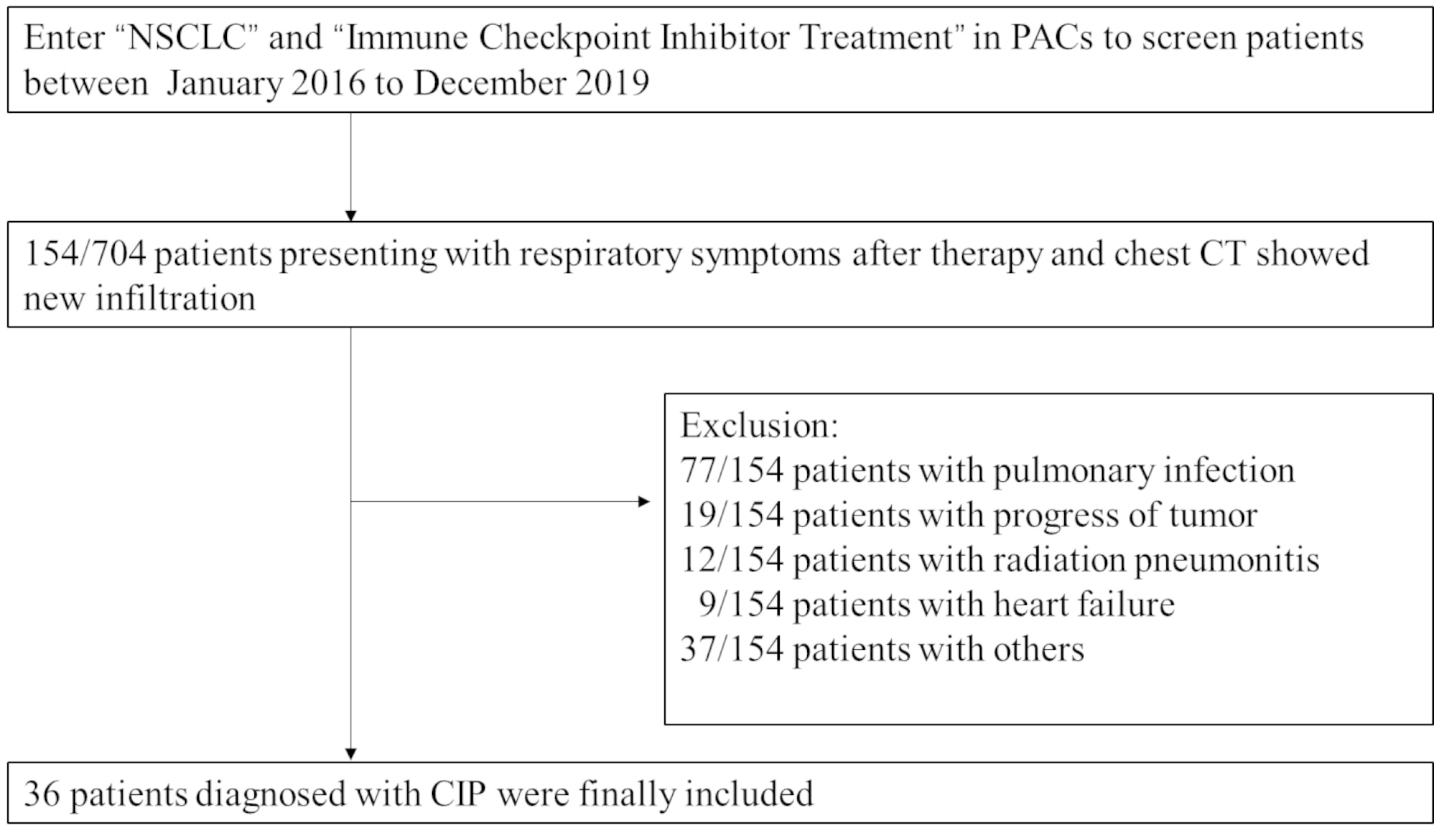
: Consort diagram of screening CIP patients

All 704 patients were screened, and 36 patients (mean age, 64.6 ± 9.8 years, 43–82 years) were included in the research. The consort diagram of screened patients was shown in Fig. 1, and the specific clinical characteristics were listed in Table 1.The incidence of CIP was 5.1%, of which 29 cases (80.6%) were male (64.6 ± 9.9 years; 43–82 years) and 7 cases (19.4%) were female (64.4 ± 10.3 years ; 45–78 years). Twenty-eight cases (77.8%) had a smoking history. There were 21 cases (58.3%) of adenocarcinoma, 12 cases (33.3%) of squamous cell carcinoma, 2 cases (5.6%) of adenosquamous carcinoma and 1 case (2.8%) of sarcomatoid carcinoma. Seven patients (19.4%) had recurrence after operation. Eight patients (22.2%) had received thoracic radiotherapy (> 6 months passed after the completion of radiotherapy). There were 10 patients (27.8%) with stage IIIB-IIIC and 26 patients (72.2%) with stage IV.

**Table 1:** Clinical Characteristics of 36 CIP Patients

Item			Varieties	No.	Frequency(%)
Age (years)		Median 64.5 (43–82) < 70 ≥ 70		27 9	75 25
Gender			Male Female	29 (64.6)* 7 (64.4.)*	80.6 19.4
Smoking			Yes No	28 8	77.8 22.2
Pathological type	Adenocarcinoma Squamous cell carcinoma Adenosquamous carcinoma Sarcomatoid carcinoma			21 12 2 1	58.3 33.3 5.6 2.8
Cancer staging			IIIb-IIIC	10	27.8
			IV	26	72.2
History of thoracic radiotherapy			Yes No	8 28	77.8 22.2
After thoracoscopic lobectomy			Yes No	7 29	19.4 80.6
Previous treatment			First-line Second-line ≥ 3 lines	16 9 11	44.4 25 30.6
Immunotherapy regimen			Single-agent Chemotherapy + immunotherapy	15 21	41.7 58.3
Immune drugs			Pembrolizumab Nivolumab Sintilimab Durvalumab Nivolumab in combination with Ipilimumab	21 8 4 2 1	58.3 22.2 11.1 5.6 2.8

* Males have an average age of 64.6 years, while females have an average age of 64.4 years

ICIs were applied to 16 patients (44.4%) as the first-line treatment, 9 patients (25%) as the second-line treatment, and the remaining 11 patients (30.6%) as the third-line and posterior-line treatment.Thirty-four patients (94.4%) were treated with PD1 inhibitor, two patients (5.5%) were treated with PDL1 inhibitor, and only one patient (2.8%) was treated with PD1 in combination with CTLA4 inhibitor. Twenty-one patients (58.3%) were treated with Pembrolizumab, 8 patients (22.2%) with Nivolumab, 4 patients (5.6%) with Sintilimab, and 2 patients (5.6%) with Durvalumab. One patient (2.8%) was treated with Nivolumab in combination with Ipilimumab. All drug doses are within the scope of the instructions.Twenty-nine patients (80.6%) survived and seven (19.4%) died. The clinical characteristics are shown in Table 1.

### Clinical manifestations

The onset time of 36 patients varied from 0.1 to 17 months, with an average onset time of 3.5 (3.5 ± 4.5) months. In accordance with the grading criteria of NCCN guidelines, 1 case (2.8%) was classified as CIP G1, 11 cases ( 30.6%) as G2, 16 cases (44.4%) as G3, and 8 cases (22.2%) as G4. There were 24 severe CIP G3-4 cases ( 66.7%) [13].

Clinical symptoms and imaging features are shown in Table 2. The most common clinical symptoms were cough in 30 cases (83.3%), shortness of breath or dyspnea in 28 cases (77.8%), fever in 10 cases (27.8%), expectoration in 10 cases (27.8%) and hemoptysis in 3 cases (8.3%). Chest pain occurred in 2 patients (5.6%) and 1 patient (2.8%) was asymptomatic with CIP G1 found on routine assessment. There were 4 cases (11.1%) complicated with rash, 3 cases (8.3%) complicated with hypothyroidism receiving L-thyroxine, 2 cases complicated with enteritis (5.6%) and 1 case (2.8%) complicated with renal insufficiency. Cardiac arrest occurred in 1 case (2.8%) and it was not clear whether the case was complicated with immunomyositis.

**Table 2:** Symptoms, Imaging Characteristics and Grade of CIP

				Examples			Proportion
Symptoms of CIP	Cough Shortness of breath or difficulty in breathing Fever Expectoration Hemoptysis Chest pain Asymptomatic				30 28 10 10 3 2 1		83.3 77.8 27.8 27.8 8.3 5.6 2.8
CIP Grade		1 2 3 4			1 11 16 8		2.8 30.6 44.4 22.2
CT Manifestations	GGO* Reticular shadows Consolidation shadows Nodule shadows Bronchitis				32 17 13 8 7		88.9 47.2 36.1 22.2 19.4
CT Imaging Characteristics			OP-like* NSIP-like* HP-like* DAD-like* Others		14 14 2 1 5		38.9 38.9 6.3 3.1 13.9
Involvement			Both lungs Single lung			28 8	77.8 22.2
Prognosis			Improvement Death			29 7	80.6 19.4

*GGO: ground-glass opacity; OP: Organizing pneumonia; NSIP: nonspecific interstitial pneumonia; HP: hypersensitiviy pneumonitis; DAD: diffuse alveolar damage

### Imaging manifestations of CIP

**Fig. 2:**
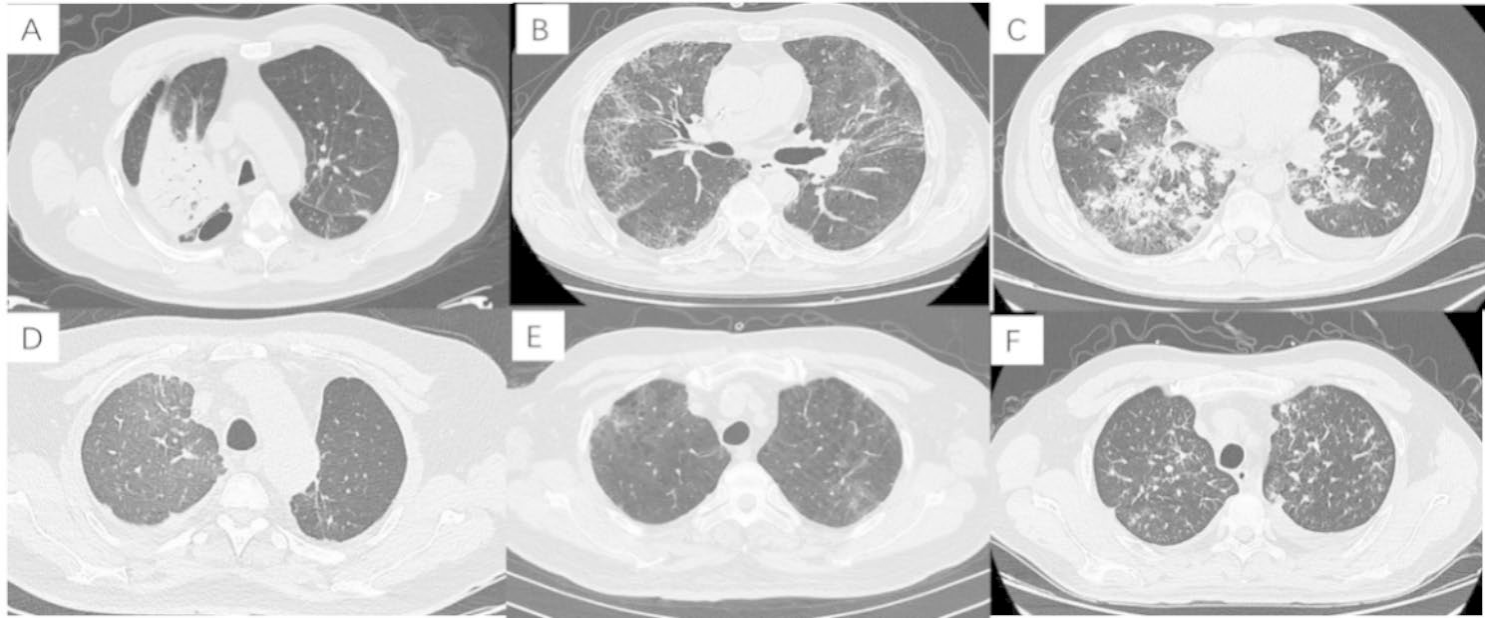
: CT characteristics of CIP: A: OP pattern: extensive consolidation in right upper lobe; B: NSIP pattern: GGO and reticular shadows in both lungs; C: AIP pattern: multiple consolidation in both lungs, reticular shadows with GGO; D: HP pattern: GGO in right upper lobe; E: HP pattern: GGO with nodule shadows in both upper lobes; F: atypical changes: bronchitic changes in both upper lobes

In 36 patients with CIP, the CT manifeststaions were as follow(Table 2): 32 cases (88.9%) with ground-glass opacity (GGO) (Fig. 2A-E), 17 cases ( 47.2%) with reticular shadows (Figs. 2B and 2C), 13 cases ( 36.1%) with consolidation shadows (Fig. 2A), 8 cases (22.2%) with nodular shadows (Fig. 2E) and 7 cases (19.4%) with bronchitis (Fig. 2E). The CT manifestations of 36 CIP patients were summarized as follows: OP-like in 14 cases (38.9%) (Fig. 2A), NSIP-like in 14 cases (38.9%) (Fig. 2B), HP-like in 2 cases (6.3%) (Fig. 2E and 2F), DAD-like in 1 case (3.1%) (Fig. 2C) and atypical imaging manifestations in 5 cases (13.9%) (Fig. 2F).

### Bronchoscopy results

Bronchoscopy was performed in 4 patients either before glucocorticosteroids (GCS) treatment. The percentage of lymphocytes count in the Bronchoalveolar lavage fluid (BALF) were 26%, 55%, 68% and 71%, respectively. Although the proportion of lymphocytes in BALF of 4 patients increased, there was no further statistical analysis due to the small number of cases.

## Treatment

The application of ICIs was discontinued or permanently discontinued in all patients, and the majority of patients (35 cases, all CIP G2 or above) received glucocorticoid therapy at specific dose and duration as shown in Table 3. Six patients were treated with gamma globulin and one patient was treated with Tocilizumab in the later stage of hormone insensitivity. All patients were not treated with other immunosuppressants.The average hormone application time was 1.9 months (1.9 ± 0.98 months, 0–4 months), and the average initial glucocorticoid dose was 85.7 mg (85.7 ± 54.5 mg, converted to prednisone dose).The average dose for CIP G2 patients was 42.3 mg (42.3 ± 11.5 mg, 25-80 mg) for an average period of 1.6 months (1.6 ± 0.45 months, 1–2 months) 82.5 mg (82,5 ± 0.81 mg, 0-200 mg) for CIP G3 patients for an average period of 2.3 months (2.3 ± 0.4 months, 1–4 months), and 162.5 mg (162.5 ± 51.8 mg, 100-200 mg) for CIP G4 patients for an average period of 1.6 months (1.6 ± 1.15 months, 0.5-4 months). The applied glucocorticoids included prednisone, methylprednisolone and dexamethasone. Six patients were initially given glucocorticoid orally and the rest 29 patients were initially given glucocorticoid intravenously.

**Table 3:** Dose and Period of Treatment of Glucocorticoid for Different CIP Grades

Grade	Oral	Intravenous	Initial dose (mg, n%)	Period of Treatment (month)
1	0	0	0	0
2	5	6	42.3 (25–80)	1.6 (1–2)
3	0	16	82.5 (50–200)	2.3 (1–4)
4	0	8	162.5 (100–200)	1.6 (0.5-4)
Total	5	30	85.7 (25–200)	1.9 (0–4)

**Fig. 3:**
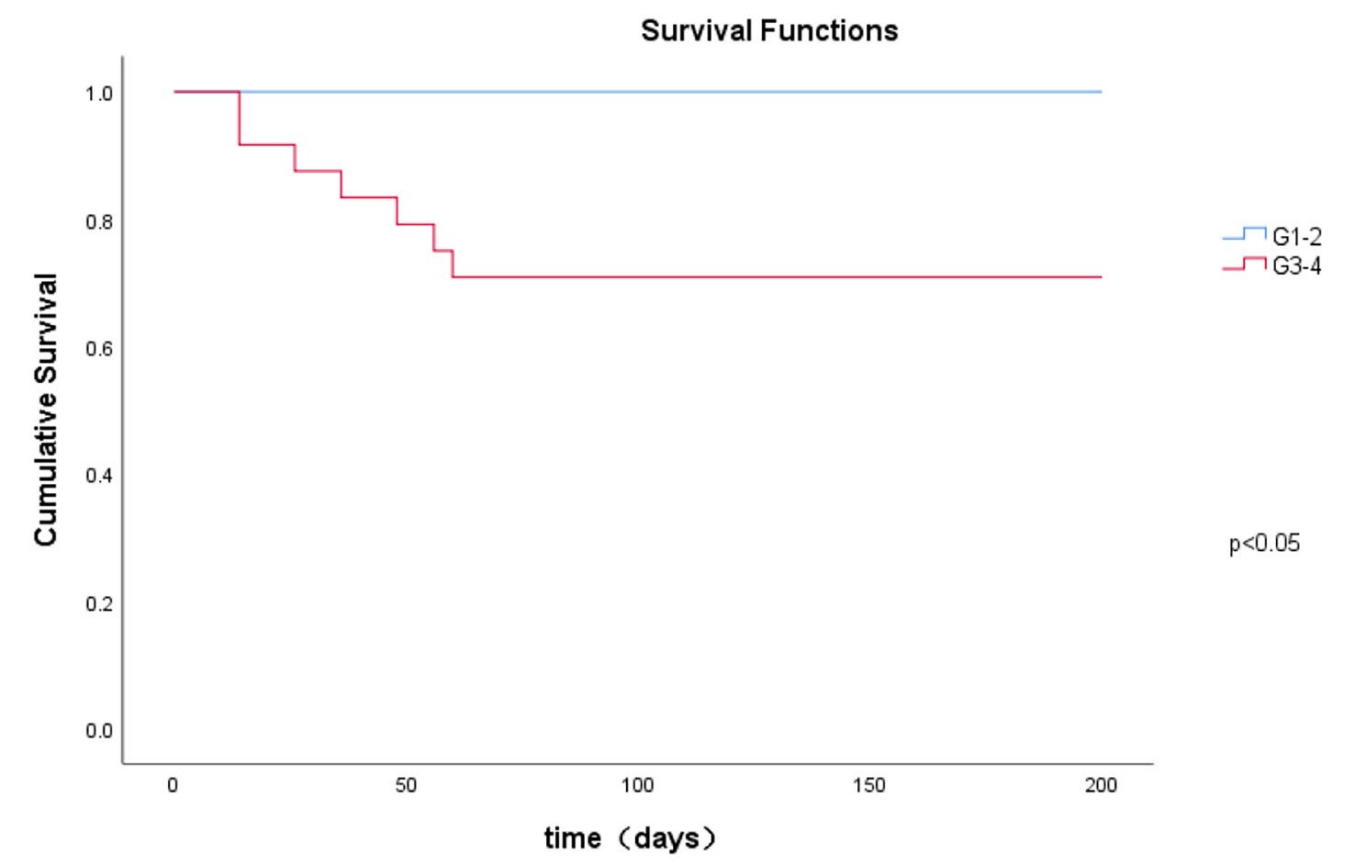
Kaplan-Meier was used to analyze the survival of patients with CIP G1-2 and G3-4

Of the total 36 patients, 7 patients died (including 1 patient who had received glucocorticoids in combination with Tocilizumab), with a mortality rate of 19.4% and 29 patients improved. There were no deaths in CIP G1-2 patients and 7 deaths occured in CIP G3-4 patients. Survival analysis showed that that survival rate in CIP G1-2 patients was significantly higher that that of CIP G3-4 patients (Fig. 3). The prognosis of patients in HP-like and OP-like groups seemed to be better than that in NSIP-like and DAD-like groups. In different imaging groups, the treatment effect of G1-2 patients was better than that of G3-4 patients, but with less number of cases, there was no statistical difference. A complete description of treatment effect is shown in Table 4.

**Table 4:** Different Imaging Types and Threapeutic Effects

Types	CIP Grading	Cases(n%)	Improvement(n%)	Death(n%)	P value
NSIP	1–2 3–4	14 5 (35.7) 9 (64.3)	11 (78.5) 5 (100) 6 (66.7)	3 (21.4) 0 3 (33.3)	0.16*
OP	1–2 3–4	14 2 (14.2) 12 (85.7)	11 (78.5) 2 (100) 9 (75.0)	3 (21.4) 0 3 (25.0)	0.43*
HP	1–2 3–4	2 1 (50.0) 1 (50.0)	2 (100) 1 (100) 1 (100)	0 0 0	-
DAD	1–2 3–4	1 (100) 0 1 (100)	0 0 0	1 (100) 0 1 (100)	-
Others	1–2 3–4	5 3 (60.0) 2 (40.0)	5 (100) 3 (100) 2 (100)	0 0 0	-
Total	1–2 3–4	36 11(30.6) 25(69.4)	29 (80.6) 11(100) 18(72.0)	7 (19.4) 0 7(28.0)	0.05*

* chi-square method ; Due to the small number of cases, the three groups (HP、DAD and others) were not statistically analyzed

Four of the 36 patients were treated again with ICIs (the same as the previous drugs). One CIP G1 patient was treated again with PD-1 after PD-1 had been discontinued for one month and imaging manifested absorption. Two CIP G1 patients and one CIP G3 patients were treated again with ICIs after improvement with hormone therapy, and no recurrent CIP occurred. See Table 5 for details.

**Table 5:** Clinical Characteristics of 4 Patients Treated with ICIs again

Patient	Pathology	Immuno-therapy Regimen (weeks)*	CIP Symp-toms	Grade	CT imaging	Treatment	Period of treat-ment
F / 67	Adenocarcinoma	Pembrolizumab (4)	None	1	GGO nodular shadows	Suspend PD-L1	Discontinuation for one month
F / 45	Adenocarcinoma	Durvalumab (7)	Cough and short-ness of breath	2	GGO	Prednisone 25 mg orally	One month
F / 78	Adenocarcinoma	Nivolumab (71)	Fever and short-ness of breath	2	GGO, reticular shadows	Methylpred-nisolone 40 mg orally	One month
M / 45	Adenocarcinoma	Nivolumab (2)	Cough and fever	3	GGO, consolida-tion	Methylpred-nisolone 80 mg intravenous-ly	Three months

* Patients 1, 2 and 3 were treated with ICI monotherapy and patient 4 (M / 45) were treated with ICI combined with immunotherapy

## Discussion

With the advancement of lung cancer therapy, ICI therapy has become a first-line treatment regimen for non-operative locally advanced and metastatic NSCLC and SCLC, of which PD1 inhibitor (PD-1 antibody and PD-L1 antibody) is the most widely used, followed by CTLA-4 inhibitor [2]. In previously published clinical trials, the incidence of CIP was about 3–5% [6, 7, 14–16], which mostly are CIP G2-3 with the incidence of G4 being only 0.67% [17].The incidence of CIP of PD-L1 inhibitor was lower than that of PD-1 inhibitor. Single-agent CTLA-4 did not increase the incidence of CIP, but when combined with PD-1 or PD-L1, it significantly increased the incidence of CIP [17]. Wang Y et al. analyzed the data of 20,128 patients from 125 clinical trials using PD-1 and PD-L1 to treat cancers from October 1, 2017 to December 15, 2018, and the results showed that among all patients, the adverse reaction with the highest incidence was fatigue (18.26%) and the incidence of CIP was 2.79%.The most common adverse reactions of patients with CIP G3 or above were fatigue (0.89%), anemia (0.78%), elevation of alanine aminotransferase (ALT) (0.75%), and the incidence of CIP was 0.67% [6]. However, most of the current data on CIP come from clinical trials, and include results in a variety of cancers. There is a lack of real-world research on locally advanced and metastatic NSCLC.

Our research data shows that the incidence of CIP is 5.1% in 706 patients. The onset time of CIP ranges from 0.1 to 17 months, and the average onset time is 3.5 months. Most patients (75.0%) belong to CIP G2-3.These results are consistent with previous studies.The most common clinical manifestations is cough, followed by shortness of breath or dyspnea. Nearly 1/3 patients have fever, and other common symptoms include expectoration and other respiratory symptoms.These symptoms are easily to be confused with respiratory infections and require more rigorous testing and treatment regimens. Some commonly used indicators of inflammation such as C-reactive protein, erythrocyte sedimentation rate, leukocyte and neutrophil percentages are also elevated in patients with CIP, so CIP cannot be well distinguished from respiratory infections [18]. Osamu Nishiyama et al. made a retrospective analysis of 12 patients with NSCLC who had undergone bronchoscopy and were pathologically confirmed as CIP, showing that the lymphocyte count in the BALF of 10 patients were higher than 20%, the neutrophil ratio was higher than 10% in 2 patients and the eosinophil ratio was higher than 10% in one patient [19]. However, in most infectious diseases, the neutrophil ratio in BALF increase, lymphocyte ratio keeps normal or decreases, and the pathogen can be found by BALF culture, NGS, etc. These methods are helpful in distinguishing CIP from pulmonary infection. Recent studies have shown that the changes of IL-6, IL-11 and other cytokines in BALF are significant for the diagnosis and prognosis of CIP [20, 21]. Therefore, bronchoscopy is very necessary to distinguish pulmonary infection from CIP.

The imaging manifestations of lung injury in CIP were not typical and varied. The basic imaging manifestations of CIP may include GGO, consolidation, fibro stripe, interlobular septal thickening, traction branchiectasia, nodule shadows and reticular shadows [22, 23]. The most common pattern of imaging combined with pathology is OP-like, followed by NSIP-, DAD-, HP-like pattern, etc. Different imaging characteristics may also be present in the same patient [22–24]. In our retrospective study, the most common types were OP- (38.9%) and NSIP-like pattern (38.9%), followed by HP-(6.3%) and DAD-like pattern (only one case, 3.3%). There were five cases of atypical imaging (13.9%). In most patients, CIP involved both lungs, and in a few cases only involved one lobe. Different imaging manifestations often indicate different degrees of disease severity, sensitivity to hormones and prognosis.Those with HP- and OP-like patterns often have a good prognosis after hormone therapy, while those with DAD-like pattern may progress rapidly, have poor sensitivity to hormone and a poor prognosis [22]. In our study, patients with HP-like pattern had a good prognosis, their symptoms improved and no deaths occured. Fourteen patients with OP-like pattern had a high severity at disease onset, among which 12 patients (85.7%) belong to G3-4, and 3 patients died after treatment. Among patients with NSIP-like pattern, 3 deaths occured and 9 (64.3%) patients belong to G3-4. The only one patient with DAD-like patten died within 1 month after receiving hormone therapy. Unfortunately, due to the small number of cases in each group, statistics did not indicate significant differences.

In patients complicated with CIP, relevant ICI drugs should be discontinued at first, and the symptoms of some G1 patients can be relieved after the discontinuation of drugs. However, for CIP patients with G2 and above, the discontinuation of drugs alone can not improve the conditions. For 70–80% of those patients, their symptoms are relieved by treatment with glucocorticoid [4]. The treatment dose and duration of glucocorticoids mainly refer to the treatment of interstitial pneumonia, and there is no definite conclusion at present. For patients with CIP G2-3, domestic experts have reached consencus and hormone therapy with an equivalent dose of 1 mg/kg/d-2 mg/kg/d prednisone is recommended, which can be used orally or intravenously (prednisone or methylprednisolon). For severe (≥ G3) CIP patients, permanent discontinuation of ICIs is required and the equivalent dose of prednisone of 2 mg/kg/d-4 mg/kg/d is recommended for more than 8 weeks [25, 26]. In our research, the average duration of hormone application was 1.9 months (0–4 months) and the average initial glucocorticoid dose was 85.7 mg (converted to prednisone dose). For patients with CIP G2, the duration of hormone application is short and the overall dose is low. For patients with CIP G3 and above who survived after treatment, hormone was applied for more than 2 months. For 7 patient who died (0.5-7 months), high dose of glucocorticoid (2 mg/kg/d-4 mg/kg/d) or even methylprednisolone (1 g/day) was ineffective. Among them, the treatmemt of one patient in combination with Tocilizumab was ineffective, and the other 6 patients were not treated with other immunosuppressants in time. For patients insensitive to these hormones, early combined application of immunosuppressants may be an effective way to reduce mortality. Long-term application of glucocorticoids in high dose inevitably leads to an increased risk of infection. Rheumatoid disease-related studies reported that the daily use of more than 20 mg of prednisone can increase the risk of infection more than five times [27].

In our research, 4 patient were retreated with ICIs, including one CIP G1 patient (self-improvement after discontinuation), two CIP G2 patients and one CIP G3 patient, and significant CIP symptoms were not observed. This may be related to the good response to glucocorticoid of latter 3 CIP patients.The paper written by Dolladille C et al., published in JAMA oncology, analyzed 24,079 cases of irAE, of which 6,123 cases were retreated with ICIs. 452 patients with relevant data were analyzed and 28.8% of patients had the same irAE again. In CIP patients, CIP recured in 34% of the 101 patients rechallenged with ICIs [28]. The choice of rechallenging patients needs to be cautious, and currently various guidelines do not provide clear recommendations for rechallenging choices. Given the findings of previous studies and our research, it is recommended that the target group of rechallenging can be partients who have stable conditions and low severity of initial CIP onset and respond well to glucocorticoid. For the target group, it is suggested that the appropriate rechallenging patients should be selected through multi-disciplinary discussion. After the reapplication of ICIs, it is necessary to monitor the symptoms associated with the irAE strictly. In case of the recurrence of CIP, ICI drugs should be withdrawn timely and glucocorticoid should be applied. If necessary, immunosuppressants should be used in combination as early as possible.

The grades of CIP are significantly related to the prognosis of patients. In our research, patients with CIP G1-2 had a good prognosis, while the mortality rate was significantly higher in patients with CIP G3-4. It is suggested that early detection and timely treatment of CIP can effectively reduce the mortality of CIP. Regular follow-up of chest CT is one of the effective methods for early detection of CIP. For the early asymptomatic patients with CIP, there is still a lack of effective and non-invasive detection methods. Studies by Lin Xinqing et al. showed that the levels of interleukin-6 (IL-6) and platelet-to-lymphocyte ratio (PLR) in CIP patients were significantly increased compared with baseline levels and close follow-up of these blood indexes can find CIP patients early [29].

As ICIs are widely applied in the treatment of NSCLC and other cancers, CIP has become a common problem facing clinicians. Further study on the clinical characteristics, treatment regimen and prognosis of CIP is helpful to improve the prognosis of patients, and to gain more treatment time for cancer patients.

## Data Availability

The datasets used in this study are available from the corresponding author upon reasonable request.
